# Privacy Personas for IoT-Based Health Research: A Privacy Calculus Approach

**DOI:** 10.3389/fdgth.2021.675754

**Published:** 2021-12-16

**Authors:** Benjamin Maus, Carl Magnus Olsson, Dario Salvi

**Affiliations:** Internet of Things and People Research Centre, University of Malmö, Malmö, Sweden

**Keywords:** citizen science, IoT-based health research, privacy calculus, privacy personas, survey

## Abstract

The reliance on data donation from citizens as a driver for research, known as citizen science, has accelerated during the Sars-Cov-2 pandemic. An important enabler of this is Internet of Things (IoT) devices, such as mobile phones and wearable devices, that allow continuous data collection and convenient sharing. However, potentially sensitive health data raises privacy and security concerns for citizens, which research institutions and industries must consider. In e-commerce or social network studies of citizen science, a privacy calculus related to user perceptions is commonly developed, capturing the information disclosure intent of the participants. In this study, we develop a privacy calculus model adapted for IoT-based health research using citizen science for user engagement and data collection. Based on an online survey with 85 participants, we make use of the privacy calculus to analyse the respondents' perceptions. The emerging privacy personas are clustered and compared with previous research, resulting in three distinct personas which can be used by designers and technologists who are responsible for developing suitable forms of data collection. These are the 1) Citizen Science Optimist, the 2) Selective Data Donor, and the 3) Health Data Controller. Together with our privacy calculus for citizen science based digital health research, the three privacy personas are the main contributions of this study.

## 1. Introduction

One way for obtaining data at scale in research is to involve citizens in the research through the voluntary engagement of citizens in scientific inquiry, also known as *citizen science*. In these initiatives, citizens act as data donors, who can be nowadays supported by digital technologies to aid scalability (1). Internet of Things (IoT) devices including mobile phones, which come equipped with all sorts of sensors and powerful cameras as well as connected devices like wearables, fitness trackers, and medical sensors have made this type of research feasible and desirable (2). These devices produce extensive, longitudinal data about our bodies (e.g., physical activity, heart rate, weight, blood oxygen saturation) and lives (e.g., social activities, use of home appliances, sleeping habits) that can be harvested for medical research (3).

Several digital platforms for citizen science exist, such as the EU-Citizen, science website, or the SPOTTERON app (1). In health-related fields, citizen science is very young and still largely controversial even if a few successful examples exist (4), for example, in the context of the Sars-Cov-2 pandemic (5). The pandemic has furthermore been acting as an accelerator of digital health technology adoption as clinical trials are being halted for fear of contagion spreading through clinical centres (6).

While IoT devices can be powerful enablers of clinical research, concerns about privacy and data protection are widely recognised (7). These concerns must be addressed both at the technology level through security measures, such as encryption and access control, and at the design level where communication with participants (8), as well as transparency and control (9), must be conveyed. Failing to do so would undermine trust in the technology and therefore limit the scope and extent of this type of research.

In order to design such trustful technologies, it is important to understand which are the typical behaviours users adopt when sharing health-related data in research, which factors affect them, and which concerns must be addressed in order to gain users' trust. While there are surveys and user studies about the interplay between data sharing and privacy [e.g., (10, 11)], as well as between mobile health and citizen science [e.g., (12, 13)], there is a lack of studies that combine these four aspects. We address this lack in our study, and as the main contribution providing a categorisation of emerging privacy attitudes and information disclosure intentionality. Driving the categorisation is the notion of *privacy personas* within the context of IoT-based health research (14), which, to the best of our knowledge, has not been previously applied in citizen science based mobile health research.

Our survey with 85 international respondents was designed based on theories on privacy calculus [cf. (15, 16)], and yielded three main personas after clustering the responses. Results from this study can be used by designers and technologists when developing new means for data collection, such as mobile phone apps or connected devices, especially in the context of health-related research. Additionally, the results are suitable for contrasting actual information disclosure in practise with the intent and underlying motivations identified in our study.

## 2. Background

### 2.1. Data Sharing and Privacy in Citizen Science Health Research

One of the core functions of citizen science is data collection and sharing. Therefore, related platforms have to deal with complex trade-offs between data quality, privacy, transparency, and trust (17). Bowser et al. define privacy in this context as “the right to manage access to voluntarily submitted personal data” and indicate the responsibility of human-computer interaction (HCI) practitioners, for example, regarding the way how platforms communicate ongoing data collection to participants (8).

Even if privacy concerns tend to be reduced among volunteers in citizen science projects (18), there is a risk of quickly decreasing their trust and motivations when not fulfilling their expectations (19) causing abandonment from the studies in which they are participating. Furthermore, as Morton and Sasse (20) indicate in their study of users' ranking of privacy, security, and trust cues, there are likely different levels of privacy concerns among segmented users. For example, age and culture (21) have a significant influence on privacy concerns and the willingness of sharing data. Zhou et al. analyse different age groups within users of mobile health apps and conclude that participants aged from 51 to 65 years were willing to use these apps but had strong privacy concerns. In contrast, participants between 18 and 28 years did not claim further privacy protection and were somewhat satisfied with the apps they were using (22).

Unlike platforms with less sensitive data collection, using citizen science apps to collect such data makes trust in the data collecting institution of paramount importance. If participants have doubts about the organisation and questions about who else might have access to the information, participants become less willing to share their data (23). The impact of trust in data sharing is based on an interplay of different factors. Trust in digital products has previously been linked to the perceived usefulness of, e.g., electronic services (24) or health-related apps (25). User studies on sharing preferences of IoT-based health data, for example, show that users are likely to share their personal information with academic researchers, medical labs, and private companies when they feel that the information requested would be useful (26) for them.

Trust can be partially gained through transparency and openness, which play a fundamental role in existing privacy frameworks for mobile health apps (9, 27). In this context, Wykes and Schueller propose the *Transparency for Trust (T4T)* principles, which are derived from experimental studies, systematic reviews, and reports of patients' concerns (28). The following four principles are suggested for the development and evaluation of mobile health apps aiming for an increased trust level:

1. **Privacy and data security**: it refers to the transparency of which data are shared from the device, like a phone or wearable, how these data are stored, and who will have access to them.
2. **Development characteristics**: it includes not only clinicians but also users in the design process of the app.
3. **Feasibility**: analysis of the usability, user experience, engagement as well as the users' security concerns.
4. **Health benefits**: it focuses on the impact on clinical outcomes.

All these principles have relevant implications in how apps for health-based research should be designed and developed and indicate the need for a deep understanding of users' privacy concerns.

### 2.2. Privacy Personas

Segmenting users based on their privacy attitudes has been researched by Alan Westin in more than 30 surveys since the 1970s (29). The “Privacy Segmentation Index” that he developed proposes three different groups of the American public who differ in their privacy concerns. Westin's first category is named *Privacy Fundamentalists* who are highly concerned about their privacy, contrary to the *Privacy Unconcerned* that are characterised by marginal concerns. The third group is the so-called *Privacy Pragmatists* with moderate concerns. Apart from the level of concern, Westin also provided representative descriptions of the three categories (30) and, thereby, created the first characteristics of privacy personas.

Westin's segmentation has been used and validated in different works (31, 32). Dupree et al. (33) provide an overview of studies that found different degrees of correlation between Westin' clusters and the tested scenarios, concluding in an “ample criticism of Westin's categorisation of users, both from a methodological perspective […] and an application/analysis perspective.”

Different sets of privacy personas have in later studies been proposed using content and clustering analysis of surveys and interviews (33) and Q-methodology (20). While Dupree et al. borrow in the case of *Fundamentalists* and *Marginally Concerned* the names of two clusters from Westin's studies, Morton and Sasse do not specifically refer to the Privacy Segmentation Index and create a new nomenclature. However, Dupree et al. observed similarities between their proposed personas named *Lazy Experts, Fundamentalists, Marginally Concerned, Technicians*, and *Amateurs* compared to Morton and Sasse's so-called *Benefits Seekers, Information Controllers, Crowd Followers, Security Concerned*, and *Organisational Assurance Seekers*. These privacy personas sets have since been used by privacy researchers in different contexts where for instance, Toresson et al. (34) base their personas on the work of Dupree et al. to create a privacy impact self-assessment app, while Murmann (35) investigate privacy notification preferences according to Morton and Sasse's personas.

## 3. Methods

To understand the privacy concerns associated with the donation of data for IoT-based health research, we designed and conducted an online survey. The survey was designed based on a model which reflects the interplay of the factors affecting our sometimes hard-to-predict information disclosure behaviour.

### 3.1. Privacy Calculus

Discrepancies between expressed privacy concerns and actual information disclosure in online environments have been investigated in several studies since the term *privacy paradox* was first mentioned (36). Early explorations indicate that users “provide significantly greater amounts of personal information than they say they will” (37). However, recent studies question this assumption and suggest a more complex interplay of privacy concerns, attitudes, and intentions (38), indicating a decision-making process where risk-benefit calculations are made (15). According to these studies, users very deliberately evaluate the perceived privacy benefits against the perceived risks. This trade-off has also been described as a *privacy calculus* (39) signifying a “function of whether the individual believes they are being given a fair exchange for […] disclosing the personal information” (40).

While most studies using privacy calculus are situated in domains, such as e-commerce or social networks (15), the approach has been also investigated within healthcare (41). For instance, Princi and Krämer (16) specifically show that there is a “potential to apply the privacy calculus in the context of IoT healthcare technology,” which helps motivate the applicability of this theory to guide our survey.

As citizen science builds on offering participants a chance to take part in research and donate data on their own terms, we consider it likely that the type of data and purpose for use of that data fits well into risk-benefit calculations. Unlike on social networks and e-commerce platforms, perceived benefits within citizen science and health research may however go beyond the notion of personal gain. Participation in citizen science is, in fact, based on the intrinsic motivation of volunteers (42). At the same time, sharing potentially sensitive health information may affect the perceived risks in great measure.

**Figure 1:**
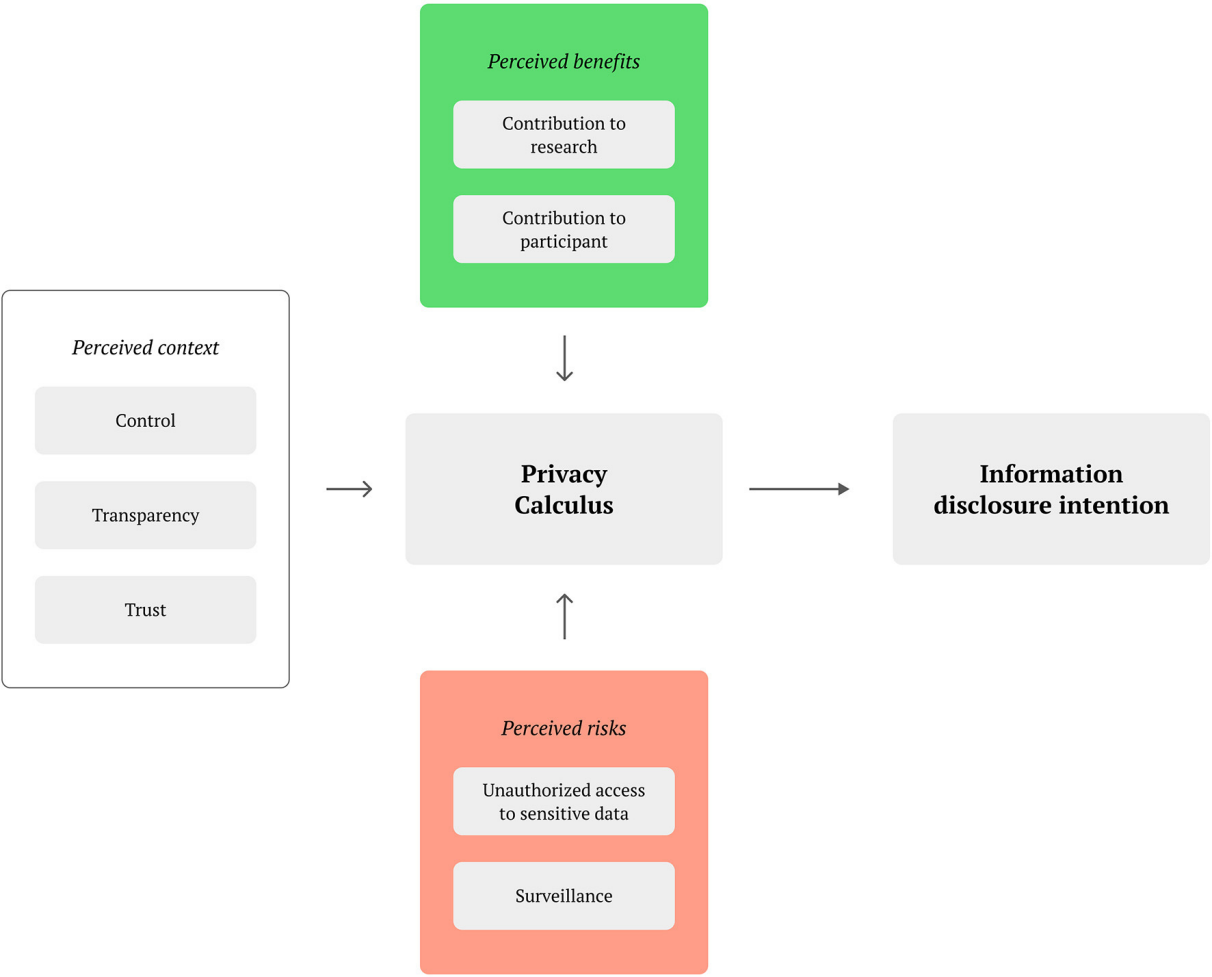
Privacy calculus in citizen science health research.

As we did not find a privacy calculus model that would fit our research context (IoT-based health-related citizen science), we decided to design a new model, partially inspired by existing literature (15, 43, 44). Our privacy calculus model (Figure 1) focuses on the evaluation of *perceived context, perceived benefits*, and *perceived risks* that participants feel in relation to donating data to a specific research project. These perceptions are therefore responsible for impacting the *information disclosure intention*, which reversely has effects on the actual information disclosure behaviour. The intention of the model is not to be generalizable or universally valid, instead, it is a tool used to understand users and cluster them in privacy personas within our research scope. The model is described in the following paragraphs.

The perceived context is influenced by the *control* over the technology that the participants perceive. This covers situations such as when perceived control over the shared data is lost (16), suitable usability design to negotiate the user group's technology experience is missing (45), as well as possible technostress visible in biological responses (46). Also belonging to the context is the perceived *transparency*, which has been highlighted as important in citizen science projects (42). Transparency may be also linked to the perceived *trust* in the institutions managing the data, particularly when sharing sensitive, health-related data with other parties.

Perceived benefits are affected by how the contribution is viewed by the participating user. This includes a *contribution to research* which can be influenced by exposure in news articles or featuring in app stores (12), but the voluntary nature of participation itself also primes participants toward openness and sharing to achieve collective benefits (18). Furthermore, *contribution to participant* concerns the value to the user for participating, such as the individual learning experience about science or about a specific health condition the user may be suffering from Rudnicka et al. (47). Participants may also be motivated by feeling recognised as citizen scientists and by being part of a community (42).

In terms of perceived risks, these largely relate to unethical data treatment. *Unauthorised access to sensitive data* is a concern for participants, especially given the health-related context (48). Importantly, data intrusion does not have to be external, as recipients of data that make use of them for different purposes than the conditions for when it was collected, also make use of data in an unauthorised way (49). The feeling of being under *surveillance* can also lead to a perceived lack of privacy when sharing large amounts of data of different kinds (50).

### 3.2. Survey Design

Our survey was based on three overall questions as guiding themes:

1. Do general privacy concerns of mobile users affect their willingness to donate data to support medical research?
2. Who do participants trust most to share their data with and how should the data be shared from their perspective?
3. How do participants perceive the impact of the app design and privacy policy on data access?

The survey addresses the first question by combining the validated quantitative 9-item Mobile Users' Information Privacy Concerns (MUIPC) scale (49) with questions about previous experiences with data donation and the willingness of participants to share their data for medical research. The MUIPC assesses privacy concerns within three main constructs: 1) “perceived surveillance,” which can be mapped to our surveillance perceived risk, 2) “secondary use of personal information,” which is strictly related to our perceived risk of unauthorised access, and 3) “perceived intrusion” which belongs to both. In addition to the MUIPC, we measured the information disclosure intention by asking participants if they were willing to share any collected data through their phones or wearables to support medical research, to which we added an open question to allow the participant to explain the reasons for their intention.

For the second general question, participants were asked to rate how comfortable they feel about sharing data with different actors and institutions such as doctors, research centres, or private companies. In addition, participants were asked for their perceptions on providing data directly to these institutions vs. through an independent third-party. These questions explore the trust category within the perceived context of our model.

The third question was addressed by asking participants about different ways to share sensitive information, authorising apps to collect data and privacy policies. This helps investigate the role of the categories of control and transparency within the perceived context. The related questions also allow participants to elaborate on the specific relevance of the design that apps and IoT technology use.

The full list of questions, with the mapping to our model, is presented in the Appendix. Prior to conducting the survey, the questionnaire was tested with two volunteers to validate that there were no obvious mistakes or parts that could cause confusion. As commonly done in citizens science research, we advertised the survey through online media and word of mouth. Participation the survey was open and anonymous. Before answering the questions, respondents had to agree to an informed consent containing a description of the project, the purpose of the study, and a description of how the survey data would be processed. In addition, all respondents had to fulfil 3 requirements, which had to be confirmed in the informed consent section: (1) 18 years or older (legal age in the country where the study was based), (2) familiarity with and regular use of a smartphone, and (3) good understanding of English (the language used in the questionnaire).

To cluster the different privacy personas of our recipients, we reviewed our questions and respondents' answers in light of the personas identified in Morton and Sasse (20). In their work, authors investigated if users of technology services could be segmented based on the type of personal information cues and privacy behaviours most important to them. In total, 69% of their study participants could be segmented into five privacy personas (20):

1. **Information Controllers**: value individual control of the collection and dissemination of their personal information.
2. **Security Concerned**: principally concerned about their own security and that of their personal information.
3. **Benefit Seekers**: focus on the benefits offered by a technology service.
4. **Crowd Followers**: follow the actions and advice of others.
5. **Organisational Assurance Seekers**: look for organisations' assurances about how they safeguard personal information entrusted to them.

## 4. Results

We start this section by describing the characteristics of our participants. This is followed by presenting the response to each of the three overall questions, which we used to guide the design of the survey.

The online survey was conducted in April 2020. In total, we received the answer from 85 respondents, the majority of which were between 18 and 35 years old (Table 1). 56% (*n* = 48) of the participants were women 41% (*n* = 35) were men and two respondents considered themselves as another gender or preferred not to answer this question. All respondents considered themselves as regular smartphone users. 64% (*n* = 54) of the participants indicated that they used their phone more than 2 h per day and only two respondents used their phone less than 30 min per day (Table 2). 60% (*n* = 51) of the participants used mainly an Android phone, while 40% (*n* = 34) were iPhone users.

**Table 1:** The age range of the participants of the survey (*n* = 85).

**Age range**	**Number of participants**
18–25 years	24 (28%)
26–35 years	37 (43%)
36–45 years	16 (19%)
46–55 years	2 (2%)
55+ years	6 (7%)

**Table 2:** Self evaluation of the participants' daily smartphone usage time (*n* = 85).

**Phone usage**	**Number of participants**
0–30 min years	2 (2%)
30–60 min	6 (7%)
60–120 min	23 (27%)
>120 min	54 (64%)

More than half of the participants (*n* = 43) used fitness- or health-related apps, such as Fitbit, Google Fit, and Samsung Health regularly but only 9% (*n* = 8) had shared data from a phone or wearable as part of a clinical study (Table 3).

**Table 3:** Current/previous use of data sharing in health/fitness apps or as part of clinical studies (*n* = 85).

	**Yes**	**No**	**Not sure**
Do you regularly use any health or fitness related app on your phone, for example to track exercise, heart rate or medication adherence? Examples are Fitbit, Apple Heart Study, GoogleFit and Samsung Health?	43 (51%)	40 (47%)	2 (2%)
Have you ever shared any data from your phone or wearable (e.g., smartwatch) as part of a clinical study?	8 (9%)	72 (85%)	5 (6%)

### 4.1. General Privacy Concerns of IoT Users Affect Their Willingness to Donate Data

Answers to the Mobile Users' Information Privacy Concerns (MUIPC) scale are reported in Table 4. The Likert scale ratings show that all the addressed categories (perceived surveillance, perceived intrusion, and secondary use of personal information) were, on average, the reason for concern to participants.

**Table 4:** Answers of the survey participants (*n* = 85) to the 9-item MUIPC scale measured on five-point Likert scales (1 = completely disagree; 5 = completely agree).

	**1**	**2**	**3**	**4**	**5**	**Average rating**
**Perceived surveillance**						
I believe that the location of my mobile device is monitored at least part of the time.	0 (0%)	4 (5%)	7 (8%)	47 (55%)	27 (32%)	4.14
I am concerned that mobile apps are collecting too much information about me.	0 (0%)	3 (4%)	19 (22%)	35 (41%)	28 (33%)	4.04
I am concerned that mobile apps may monitor my activities on my mobile device.	1 (1%)	3 (4%)	23 (27%)	45 (53%)	13 (15%)	3.78
**Perceived intrusion**						
I feel that as a result of using mobile apps, others know more about me than I am comfortable with.	0 (0%)	8 (9%)	26 (31%)	32 (38%)	19 (22%)	3.73
I believe that as a result of using mobile apps, too much personal information is available to others than I am comfortable with.	0 (0%)	9 (11%)	21 (25%)	36 (42%)	19 (22%)	3.76
I feel that as a result of using mobile apps, information about me is out there that, if used, will invade my privacy.	1 (1%)	8 (9%)	28 (33%)	30 (35%)	18 (21%)	3.66
**Secondary use of personal information**						
I am concerned that mobile apps may use my personal information for other purposes without notifying me or my authorisation.	2 (2%)	7 (8%)	12 (14%)	37 (44%)	27 (32%)	3.94
When I authorise apps to use personal information, I am concerned that these apps may use my information for other purposes.	2 (2%)	8 (9%)	13 (15%)	37 (44%)	25 (29%)	3.88
I am concerned that mobile apps may share my personal information with other institutions without my authorisation.	1 (1%)	13 (15%)	16 (19%)	30 (35%)	25 (29%)	3.76

Table 5 shows the participants' information disclosure intention in the context of IoT-based medical research. In total, 67% (*n* = 57) were willing to share data like location, steps, calories, and heart rate in medical studies, while 24% (*n* = 20) were not sure and 9% (*n* = 8) were not willing to share any data. No significant correlation was found between the average MUIPC score and the willingness to disclose information (Pearson's correlation coefficient = –0.15).

**Table 5:** Information disclosure intention measured with the question “Would you be willing to share any data collected through your phone or wearable device (e.g., location, steps, calories, heart rate) as part of participating in a clinical study?” (*n* = 85).

**Answer**	**Number of participants**
Yes	57 (67%)
Not sure	20 (24%)
No	8 (9%)

In total, 71 participants answered the open question about the reason why they were willing/not sure/not willing to share data as part of clinical studies. Our discussion in Section 5 quotes the most representative answers for all the three cases.

### 4.2. Participants Trust of Data Recipients

Table 6 shows that respondents indicated to have more privacy concerns for social media, banking or photo apps than for health and fitness apps or for data donation platforms.

**Table 6:** Answers to the question “How concerned are you about privacy when it comes to the following types of apps?” (*n* = 85).

	**Very concerned**	**Somewhat concerned**	**Neither concerned nor unconcerned**	**Somewhat unconcerned**	**Not concerned at all**
Social media apps (e.g., Facebook, TikTok, LinkedIn)	21 (25%)	37 (44%)	11 (13%)	12 (14%)	4 (5%)
Fitness apps (e.g., Google Fit, Strava, Endomondo)	6 (7%)	28 (33%)	19 (22%)	24 (28%)	8(9%)
Banking/wallet apps (e.g., Swish, PayPal, Apple Pay)	37 (44%)	14 (16%)	11 (13%)	16 (19%)	7 (8%)
Medical apps (e.g., Evergreen, Epocrates, MDLive)	9 (0%)	24 (0%)	31 (0%)	14 (0%)	7 (0%)
Photos apps (e.g., Google Photos, PIxlr)	18 (21%)	31 (36%)	15 (18%)	13 (15%)	8 (9%)
Apps for sharing data in clinical studies (e.g., Mobistudy, Ohmage, MyCap)	6 (7%)	19 (22%)	35 (41%)	16 (19%)	9 (11%)

More specifically in the health field, participants were asked how comfortable they would feel to share any of their mobile health data with different types of organisations (Table 7). Respondents felt most comfortable with sharing their data with clinicians and doctors, public health institutions, and research centres or universities.

**Table 7:** Answers to the question “How comfortable would you feel to share any of your mobile health data with the following?” (*n* = 85).

	**Very uncomfortable**	**Somewhat uncomfortable**	**Neither comfortable nor uncomfortable**	**Somewhat comfortable**	**Very comfortable**
A hospital/clinic/ doctor	3 (4%)	10 (12%)	9 (11%)	37 (44%)	26 (31%)
A public health institution (e.g., health authority)	4 (5%)	15 (18%)	15 (18%)	32 (38%)	19 (22%)
A research centre or university	3 (4%)	7 (8%)	15 (18%)	28 (33%)	32 (38%)
A private company	33 (39%)	28 (33%)	14 (16%)	8 (9%)	2 (2%)
A non-profit company or charity	8 (9%)	19 (22%)	27 (32%)	21 (25%)	10 (12%)

Regarding the involvement of third parties, 71% (*n* = 60) of the participants preferred providing institutions with their data in a direct way and 18% considered either a third, independent party (*n* = 8) or both institutions and third parties (*n* = 8) as their preference.

### 4.3. Impact of App Design and Privacy Policy on Data Access

The role of privacy policies is explored in Table 8, where it is shown that the majority of the participants (*n* = 50) rarely or never read nor understand the privacy policies of the apps they install. The lack of understandings go along with a low level of trust in those policies (Table 9), as about half of the respondents (*n* = 43) do not feel confident to rely on the privacy policy of apps to know which data is accessed.

**Table 8:** Perception of privacy policies (*n* = 85).

	**Never**	**Rarely**	**Sometimes**	**Most of the time**	**Always**	**Not sure**
How often do you read the privacy policies of the apps you install?	26 (31%)	34 (40%)	20 (24%)	1 (1%)	4 (5%)	0 (0%)
Do you usually find privacy policies easy to understand?	24 (28%)	31 (36%)	17 (20%)	5 (6%)	0 (0%)	8 (9%)

**Table 9:** Answers to the question “In order to know what data is accessed and by which app, how confident would you be with the following approaches?” (*n* = 85).

	**Not confident at all**	**Not confident**	**Somewhat confident**	**Confident**	**Very confident**
I trust the privacy policy of each app.	9 (11%)	34 (40%)	27 (32%)	15 (18%)	0 (0%)
Each app provides information about how data is accessed and used.	5 (6%)	23 (27%)	39 (46%)	16 (19%)	2 (2%)
The operating system (Android or iOS) or a third-party app informs me how installed apps use my data.	10 (12%)	14 (16%)	32 (38%)	25 (29%)	4 (5%)

Another objective of the survey was to find out how participants expected apps to operate when collecting data within a health-related citizen science project. Most of the users felt either comfortable (*n* = 44) or very comfortable (*n* = 14) if the operating system of their phone asked them for permission in order to authorise the app to access sensitive information. Respondents expressed lower confidence about third-party systems or apps asking for permission, in fact, 31% (*n* = 26) felt comfortable or very comfortable (7% *n* = 6) letting them authorise other apps. Not a single participant felt comfortable with the option that apps could directly access sensitive information without any permission (Table 10).

**Table 10:** Answers to the question “If an app needs to access sensitive information from your phone (for example, your geographical position), what option would you feel more comfortable with?” (*n* = 85).

	**Not comfortable at all**	**Uncomfortable**	**Somewhat comfortable**	**Comfortable**	**Very comfortable**
The app directly accesses the data without needing my permission.	48 (56%)	32 (38%)	5 (6%)	0 (0%)	0 (0%)
The phone's operating system (Android or iOS) asks my permission to authorise the app.	1 (1%)	5 (6%)	21 (25%)	44 (52%)	14 (16%)
A third-party system or app asks my permission to authorise the app.	8 (9%)	22 (26%)	23 (27%)	26 (31%)	6 (7%)

Regarding how frequent the authorisation process should be, the preferences of the participants varied. Only 13% (*n* = 12) wanted to authorise the app only one time, almost half of the respondents (*n* = 42) preferred to authorise the app each time it required data and 45% (*n* = 38) of the participants would like to authorise the app only one time and be remembered about the data collection after a certain amount of time.

## 5. Discussion

In order to analyse our results we will here link these with the privacy calculus model and related literature. The quotes provided in this section have been selected as representative of the 71 respondents that provided qualitative elaboration, and illustrate the analysis we have made of such answers. The discussed results are then clustered in order to define a set of privacy persona for technology-aided health research. Reflections on the limitations of our study end the section.

### 5.1. Perceived Context

In terms of perceived context, we clearly see the relevance of control, transparency, and trust for our respondents as they determine their willingness to participate in studies. A lost sense of control (16) is the most common concern expressed, where the recipient of the data as well as data, security, are frequently mentioned as important.

“*Depends on the info if you could choose specifically what data sharing would do it. If it is not clear, no.”*

“*Not sure about who collects my data and the use of them.”*

Participants clearly wanted apps to ask for permission before accessing data on their phone, either through the operating system or, to a smaller extent, through third-party apps. A frequent authorisation process was considered more desirable than a one-time approach. We also see that authorisation carries overlapping elements with transparency. For example, participants express that their willingness to share data is affected by the understanding of who does what with the data and for which purpose. The operating system or a third party app were identified as the preferred method for authorising data access to apps.

Privacy policies subsequently appear to only partially satisfy the contextual needs of transparency and trust (42). Our results show that they are rarely read, also because they are hard to understand. Similar to the findings of previous studies (51), the length of the agreements and the perceived cost vs. the benefit of reading them is an issue, but importantly the legal language used is often problematic for average users.

Our findings thus support the need for transparency enhancing tools, especially if they offer a high degree of customisation in order to accommodate different users, profiles (35). It may be hypothesised that allowing participants to review data prior to sharing could be a trust-building mechanism for users who are not comfortable trusting data recipients. Further studies assessing the design and implementation of such mechanisms, for example, based on Nebeker et al. (52), would be needed to validate this.

As detailed in the privacy calculus, this brings us to the relevance of trust. Here, our respondents clearly show less concern with fitness, medical or research apps, and have less concerns when sharing their data with clinicians, healthcare, and research institutions rather than private companies. Institutional trust is nonetheless not enough and each context for data donation is of relevance to some participants.

“*It depends on the goals of the study and who is conducting it. (Trust levels would have to be high!) But if I believe I can contribute to something meaningful with something this small, I would.”*

“*I am afraid that this data will be used for commercial reasons or that the wrong person will get access to my data. Moreover, I am afraid that insurance companies will use the data to personalize insurances.”*

This confirms what was found in Lupton (53) where trust dimensions were identified as closely associated with sharing self-tracked data. In their study, context stood out as strongly linked with the willingness to share, as personal information was commonly viewed as an intimate social experience.

### 5.2. Perceived Benefits

The dominating perceived benefit our respondents see for participation is to help contribute to research that may improve healthcare for all. Our survey thus shows that motivations for sharing data were similar to the findings in citizen science studies that were not related to health (18). These include the perceived benefits of contribution to research and science, which was also amplified by the perception of low effort or low risk to some respondents.

“*I think it is important for science to have enough data to find important results. I would hope that there will be clinical improvements through my sharing of data.”*

“*But if I believe I can contribute to something meaningful with something this small, I would.”*

Half of the respondents reported using health and fitness apps, which may indicate the fact that they get some sort of personal benefit from them. However, somewhat surprising to us, only one participant explicitly recognises the potential to contribute to personal conditions, as previously identified relevant by Rudnicka et al. (47).

“*I've participated in an allergy study before in an attempt to understand my own health issues in order to improve them. Contributing data is a way to help oneself or someone like yourself.”*

The reason why personal benefit was not a recurring theme in the open text answers may be justified by the fact that only 8 respondents declared to have had an experience of data sharing in clinical studies, or simply by their prioritising contribution to research over themselves. What we do see is that collective benefits (18) are important to be clear about for prospective candidates to determine interest in participation.

### 5.3. Perceived Risks

As users are presented with new technology, previous experiences and general attitudes have an impact on the privacy calculus process. Looking at *perceived risks*, the results of the MUIPC questionnaire show that all our respondents had general privacy concerns in relation to sharing data through mobile phones (average rating to all MUIPC questions was higher than the “neutral” value 3). Both categories of surveillance and unauthorised access to data were identified as concerning. Lack of trust appears to be the main driver for this, and quotes confirm both concerns for external parties accessing the data (48) and the use of data for other purposes than those stated in the research (49).

“*I don't trust the companies that are using or selling the data.”*

“*I am afraid that this data will be used for commercial reasons or that the wrong person will get access to my data. Moreover, I am afraid that insurance companies will use the data to personalize insurances.”*

Interestingly, while our respondents note that they may be willing to donate data for clinical use, they may still feel somewhat uncertain as to the privacy aspects of doing so. This may also extend to very specific data types which to some are perceived as particularly sensitive like geographical position.

“*If it would be for clinical use only, I might-but would feel unsafe in terms of privacy: is the data traceable to me? Will they only access the health data or other data I have on my phone? Will it be further used for insurances or other third parties?”*

“*I think it depends on what the trial is about and how (GPS) data will be processed.”*

While secondary use of data may be straightforward to avoid by simply not reusing data in other contexts, some have argued that it is short-sighted to keep data in isolated silos (54). Arguments to this end include the growth of the research community and conforming to demands from funding agencies.

### 5.4. Privacy Personas

To cluster the different privacy personas of our recipients, we started by reviewing our questions and respondents, answers in light of the Morton and Sasse (20) personas. Given the difference in focus between their study and ours, we expected some mismatches and a need for adaptation. Additionally, as only 69% of the Morton and Sasse (20) study participants could be segmented into these privacy personas, we also expected that all our participants may not fit into clearly defined clusters. Rather than force clustering, we, therefore, focused on those that clearly could be seen and that held strong similarities to those identified by Morton and Sasse.

We clustered the answers provided to the single choice questions as well as the qualitative data provided through the open answer. Following this process, we identified three privacy personas as relevant within IoT-based health research using citizen science (Table 11). The names, *Citizen Science Optimist, Selective Data Donor*, and *Health Data Controller*, are rooted in the naming conventions of Morton and Sasse (20) given the role their research played in our analysis, but have been contextualised to our application area.

**Table 11:** Proposed privacy personas in IoT-based health research using citizen science.

	**Citizen science optimist**	**Selective data donor**	**Health data controller**
*Selected quote from study*	“Because it's a little bit of information from me, but it would help to improve clinical studies a lot.”	“It depends a lot on who is conducting the study. I would only trust a university or a university hospital.”	“Depends on the information. If you could choose specifically what data is going to be shared, I would do it. If it is not clear, no.”
*Description*	Citizen Science Optimists are characterised by users who perceive data donation as something small and manageable. Even if they are aware of potential privacy risk, they are usually focused on the greater good of supporting researchers and scientists.	Selective Data Donors refer to users who evaluate the institution that conducts a medical study very carefully before taking part in any data donation. They value reading the detailed privacy policy of the app/study.	Data Controllers are users who try to handle their data carefully, e.g., turn off their GPS regularly. They are willing to donate their data but require additional tools to experience a higher security sensation.
*Connection to Morton and Sasse (2014)*	Related to *Benefit Seekers* but instead of being focused on their own benefits, altruistic thinking is predominant. However, few comments from the survey show that there might be cases where Citizen Science Optimists are less altruistic (e.g., “Contributing data is a way to help oneself or someone like yourself.”).	Similar to *Organisational Assurance Seekers* who validate mainly the organisation before using any technology. Since health data might contain sensitive information, the evaluation of the institution might be even more crucial than in other categories.	Similar to *Information Controllers* who appreciate high control over their information and data at any time which also includes the temporality of their data, i.e., deleting their data when they are not required anymore to avoid traceability.

Citizen Science Optimist makes up the majority of our cohort (46% of our respondents, *n* = 39). They are users who focus on the greater good and the benefits of research more than privacy concerns. They are characterised by being more likely to share their data in clinical studies (100%), compared with Selective Data Donors (83%) and Health Controllers (14%). The Citizen Science Optimist further holds a lower mean MUIPC score (3.72 on average, compared to 4.02 and 4.03, respectively), who are unconcerned or somewhat concerned about using apps in clinical research (41% compared with 0% and 14%), as well as more likely to accept sharing their data with a private company (18 vs. 0 and 7%).

Selective Data Donors constitute the smallest group (7%, *n* = 6) of our participants. Their disclosure intention can be high (83%), but it depends on the trust they give to the institutions collecting or processing their data. They are characterised by being less comfortable sharing their data with any actor, public, or private (67 vs. 54% of Citizen Science Optimists and 43% of Health Data Controllers) and keener to involve an independent third party when sharing their data (17% positive vs. 8 and 7%).

In our cohort, Health Data Controllers correspond to 16% (*n* = 14) of the participants. They are users who pay particular attention to controlling the data sharing process, for example by setting permissions on their phones. They are less likely to share their data for research (14%), and when they do, they prefer to share data with doctors or researchers directly (86% compared with 74% of Citizen Science Optimists and 67% of Selective Data Donors). The Health Data Controllers are also most likely to be comfortable with providing apps access to their data if either the operating system or a third party app acts as access control (79%, vs. 72 and 50%), or informs about how the data is used (86%, vs. 79 and 83%).

In our survey, we could not identify Morton's *Security Concerned* or *Crowd Follower* among our participants. As previously recognised (20), users similar to the *Security Concerned* personas are less likely to participate in citizen science initiatives, which together with the voluntary recruitment approach likely means this category is less likely to be found. Trace patterns of the *Crowd Follower* persona could be seen but was deemed to be negligible in the analysed data and therefore not included. The persona is plausible to be found in larger data sets than ours but appears to likely be less common than the three we identified.

Overall, our privacy personas are applicable to IoT- and mobile- supported health research. They are in particular applicable for the purpose of understanding willingness to share data for research purposes in citizen science projects, for example, when designing studies, communicating privacy policies, or developing tools for data collection and sharing.

### 5.5. Limitations and Future Work

Even if a robust number of respondents participated in the survey (*n* = 85), we did not aim at the statistical significance and therefore did not test any hypothesis through statistical means. Further validation with larger cohorts would therefore be relevant to fully establish the proposed personas. We also recognise that our cohort was young, as 71% of the respondents were below 35 and relatively tech-savvy, as 90% of our respondents use the phone for 1 h or more every day. Results may therefore be different on a more general population.

For example, differences in data sharing willingness may arise depending on specific health conditions that respondents may have, as patients with severe or chronic conditions may be more motivated than healthier counterparts. Hypothesis driven testing in larger cohorts could also allow validation of the observed characteristics within our privacy personas, the prevalence of the personas themselves, as well as perhaps the Crowd Follower persona, which we could not confirm but saw traces of.

Even if transparency emerged as an important component in our results, we did not explore the impact of specific nuances related to informed consent. Such informed consent is often mandatory in health research, given the sensitivity of the data requested from participants. Recent research by Nebeker et al. (52), for example, identify the role of content and delivery in establishing meaningful informed consent within Digital Health. Future research could assess links between Privacy Personas identified in our work with the guidelines offered by Nebeker et al., in order to shed light on how to effectively deliver personalised and transparent consent processes. Based on these results, concrete guidelines and design patterns for health-related technologies could also be designed based on the privacy personas and relevant legislation such as the General Data Protection Regulation (GDPR).

Finally, it is also crucial to highlight that privacy concerns expressed by users may differ from their actual data sharing behaviour (55). New studies should compare the data disclosure intention with data disclosure actual behaviour to validate similarities and identify any discrepancies. In this regard, even if surveys can help understand general attitudes, further methods like experiments, user studies, or user testing are necessary to understand behaviour patterns fully.

## 6. Conclusions

In this study, we report on a survey of privacy, trust, and data access concerns associated with conducting citizen science based health research using IoT technology. While extant research reports on the interplay of data sharing and privacy [e.g., (10, 11)], as well as the intersection between mobile health and citizen science [e.g., (12, 13)], our research addresses the gap in studies that combine these four aspects.

Our main contributions include developing a privacy calculus for this form of health research, which after applying it as a lens to our survey yielded three distinct privacy personas: (1) Citizen Science Optimist, (2) Selective Data Donor, and (3) Health Data Controller. We provide the characteristics that define each persona which is relevant for designers of studies, and technologists developing data collecting IoT services, associated with health-related research.

We also contribute by outlining several promising directions for future work based on our findings. This includes relying on our privacy personas when exploring the impact of different strategies for informed consent processes and content, developing guidelines and design patterns aligned with legislative directives, and comparing the expressed perceptions on privacy with actual data sharing behaviour.

## Data Availability Statement

The raw data supporting the conclusions of this article will be made available by the authors, without undue reservation.

## Ethics Statement

Ethical review and approval was not required for the study on human participants in accordance with the local legislation and institutional requirements. The patients/participants provided their written informed consent to participate in this study.

## Author Contributions

BM has conducted the survey, supervised by DS with advice from CO in terms of design, execution, and analysis. All authors shared the aspects of writing the paper and prepared the final work for the publication.

## Funding

This project was partially funded by the Swedish Knowledge Foundation through the IoT and People research profile (Dnr. 20140035).

## Conflict of Interest

The authors declare that the research was conducted in the absence of any commercial or financial relationships that could be construed as a potential conflict of interest.

## Publisher's Note

All claims expressed in this article are solely those of the authors and do not necessarily represent those of their affiliated organizations, or those of the publisher, the editors and the reviewers. Any product that may be evaluated in this article, or claim that may be made by its manufacturer, is not guaranteed or endorsed by the publisher.
