# Supplementary material for: Privacy Personas for IoT-Based Health Research: A Privacy Calculus Approach
Source: Front Digit Health. 2021 Dec 16;3:675754. doi: 10.3389/fdgth.2021.675754 (PMC8716597; doi:10.3389/fdgth.2021.675754)
Supplement: Supplementary file 1 [file Table_1.pdf]

Supplementary Material

1 APPENDIX

Table S1: Overview of the survey question/items

**Questions/items in our survey**	**Related items in our privacy calculus model**
Characteristics of the participants	
**What is your age group?**<br>1) 18-25<br>2) 26-35<br>3) 36-45<br>4) 46-55<br>5) 55+	
**What is your gender?**<br>1) Woman<br>2) Man<br>3) Other/Prefer not to say	
**How much time do you use your smartphone per day on average?**<br>1) 0-30 minutes<br>2) 30-60 minutes<br>3) 1-2 hours<br>4) More than 2 hours	
**What kind of smartphone do you mainly use?**<br>1) iPhone<br>2) Android<br>3) Other<br>4) None	

Continued on next page

Table S1 – *Continued from previous page*

Questions/items in our survey	Related items in our privacy calculus model
**Do you regularly use any health or fitness related app on your phone, for example to track exercise, heart rate or medication adherence? Examples are Fitbit, Apple Heart Study, GoogleFit and Samsung Health.**<br>1) Yes<br>2) No<br>3) Not sure	Perceived Benefits: *Contribution to participant*
**Have you ever shared any data from your phone or wearable (e.g. smartwatch) as part of a clinical study?**<br>1) Yes<br>2) No<br>3) Not sure	Perceived Benefits: *Contribution to research*
Do general privacy concerns of mobile users affect their willingness to donate data to support medical research?	
**To what extent do you agree to the following statements? (based on 9-item MUIPC scale (?))**	
I believe that the location of my mobile device is monitored at least part of the time.	Perceived risks: *Surveillance*
I am concerned that mobile apps are collecting too much information about me.	Perceived risks: *Surveillance*
I am concerned that mobile apps may monitor my activities on my mobile device.	Perceived risks: *Surveillance*
I feel that as a result of using mobile apps, others know more about me than I am comfortable with.	Perceived risks: *Surveillance*
I believe that as a result of using mobile apps, too much personal information is available to others than I am comfortable with.	Perceived risks: *Surveillance*
I feel that as a result of using mobile apps, information about me is out there that, if used, will invade my privacy.	Perceived risks: *Unauthorised access to sensitive data*
I am concerned that mobile apps may use my personal information for other purposes without notifying me or my authorisation.	Perceived risks: *Unauthorised access to sensitive data*

Continued on next page

Table S1 – *Continued from previous page*

**Questions/items in our survey**	**Related items in our privacy calculus model**
When I authorise apps to use personal information, I am concerned that these apps may use my information for other purposes.	Perceived risks: *Unauthorised access to sensitive data*
I am concerned that mobile apps may share my personal information with other institutions without my authorisation.	Perceived risks: *Unauthorised access to sensitive data*
**Would you be willing to share any data collected through your phone or wearable device (e.g. location, steps, calories, heart rate) as part of participating in a clinical study?**<br>1) Yes<br>2) Not sure<br>3) No	*Information disclosure intention*
**Could you briefly tell why?**	All
Whom do participants trust most to share their data with and how should the data be shared from their perspective?	
**How concerned are you about privacy when it comes to the following types of apps?**<br>1) Social media apps<br>2) Fitness apps<br>3) Banking/wallet apps<br>4) Medical apps<br>5) Photos apps<br>6) Apps for sharing data in clinical studies	Perceived Context: *Trust*
**How comfortable would you feel to share any of your mobile health data with the following?**<br>1) A hospital/clinic/doctor<br>2) A public health institution (e.g. health authority)<br>3) A research centre or university<br>4) A private company<br>5) A non-profit company or charity	Perceived Context: *Trust*

Continued on next page

Table S1 – *Continued from previous page*

Questions/items in our survey	Related items in our privacy calculus model
**Would you rather provide your data directly to researchers (university or company), or would you prefer giving it to a third, independent party that makes them available to researchers?**<br>1) I'd rather share my data directly with researchers<br>2) I'd prefer using a third, independent party<br>3) Both<br>4) None<br>5) Not sure	Perceived Context: *Trust*
How do participants perceive the impact of the app design and privacy policy on data access?	
**In order to know what data is accessed and by which app, how confident would you be with the following approaches?**<br>1) I trust the privacy policy of each app.<br>2) Each app provides information about how data is accessed and used.<br>3) The operating system (Android or iOS) or a third-party app informs me how installed apps use my data.	Perceived Context: *Transparency*
**When an app accesses sensitive information from your phone, would you rather**<br>1) Authorise the app only once.<br>2) Authorise the app each time it requires the data.<br>3) Authorise the app and be reminded after a certain amount of time (e.g. a month)	Perceived Context: *Control*
**If an app needs to access sensitive information from your phone, what option would you feel more comfortable with?**<br>1) The app directly accesses the data without needing my permission.<br>2) The phone's operating system (Android or iOS) asks my permission to authorise the app.<br>3) A third-party system or app asks my permission to authorise the app	Perceived Context: *Control*

Continued on next page

Table S1 – *Continued from previous page*

**Questions/items in our survey**	**Related items in our privacy calculus model**
**How often do you read the privacy policies of the apps you install?**<br>1) Always<br>2) Most of the time<br>3) Sometimes<br>4) Rarely<br>5) Never<br>6) Not sure	Perceived Context:<br>*Transparency*
**Do you usually find privacy policies easy to understand?**<br>1) Always<br>2) Most of the time<br>3) Sometimes<br>4) Rarely<br>5) Never<br>6) Not sure	Perceived Context:<br>*Transparency*
